# HIV and STI Prevalence and Injection Behaviors Among People Who Inject Drugs in Nairobi: Results from a 2011 Bio-behavioral Study Using Respondent-Driven Sampling

**DOI:** 10.1007/s10461-014-0936-3

**Published:** 2014-11-15

**Authors:** Waimar Tun, Meredith Sheehy, Dita Broz, Jerry Okal, Nicholas Muraguri, H. Fisher Raymond, Helgar Musyoki, Andrea A. Kim, Mercy Muthui, Scott Geibel

**Affiliations:** 1 HIV and AIDS Program, Population Council, 4301 Connecticut Avenue, NW, Suite 280, Washington, DC 20008 USA; 2 Population Council, New York, NY USA; 3 HHS-Centers for Disease Control and Prevention, Division of Global HIV/AIDS, Center for Global Health, Atlanta, GA USA; 4 Division of Global HIV/AIDS, Center for Global Health, Atlanta, GA USA; 5 Population Council, Nairobi, Kenya; 6 National AIDS and STD Control Programme, Nairobi, Kenya; 7 San Francisco Department of Health, San Francisco, CA USA; 8 HHS-Centers for Disease Control and Prevention, Division of Global HIV/AIDS, Center for Global Health, Nairobi, Kenya; 9 Division of Global HIV/AIDS, Center for Global Health, Nairobi, Kenya

**Keywords:** HIV prevalence, STI prevalence, People who inject drugs, Kenya, Integrated biobehavioral, Surveillance survey, Respondent-driven sampling

## Abstract

There is a dearth of evidence on injection drug use and associated HIV infections in Kenya. To generate population-based estimates of characteristics and HIV/STI prevalence among people who inject drugs (PWID) in Nairobi, a cross-sectional study was conducted with 269 PWID using respondent-driven sampling. PWID were predominantly male (92.5 %). An estimated 67.3 % engaged in at least one risky injection practice in a typical month. HIV prevalence was 18.7 % (95 % CI 12.3–26.7), while STI prevalence was lower [syphilis: 1.7 % (95 % CI 0.2–6.0); gonorrhea: 1.5 % (95 % CI 0.1–4.9); and Chlamydia: 4.2 % (95 % CI 1.2–7.8)]. HIV infection was associated with being female (aOR, 3.5; *p* = 0.048), having first injected drugs 5 or more years ago (aOR, 4.3; *p* = 0.002), and ever having practiced receptive syringe sharing (aOR, 6.2; *p* = 0.001). Comprehensive harm reduction programs tailored toward PWID and their sex partners must be fully implemented as part of Kenya’s national HIV prevention strategy.

## Background

Injection drug behaviors have been recognized as key facilitators of HIV transmission since the beginning of the epidemic [1–3]. In 2010, there were an estimated 15.9 million (ranging from 11 to 21.2 million) people who injected drugs (PWID) globally, with one in five estimated to be HIV-positive [2]. While Southeast and East Asia have the largest number of PWID [4], recent evidence shows an increase in injection drug use and associated HIV infections in sub-Saharan Africa, where the burden of HIV is already the highest in the world [5–16]. Despite a lack of comprehensive evidence, it is clear that PWID in sub-Saharan Africa are at great risk for acquiring and transmitting HIV in the context of few prevention interventions targeted toward this population and lack of capacity to handle key populations at risk for HIV such as men who have sex with men, sex workers, and PWID, in the current state of the burdened healthcare system.

African countries with significant HIV epidemics such as Kenya, where general population prevalence is 5.6 % [17], are only now in the beginning stages of understanding the role of key populations with higher risk for exposure to HIV infection in their epidemic, including PWID. In Kenya, use of injected heroin reportedly increased in the late 1990s with the availability of ‘white crest’ from Thailand [18]. While white crest replaced ‘brown sugar’, a lower grade of heroin, user habits shifted from inhalation of the vapor to injecting [18]. The number of PWID has been a point of contention among researchers, however, the consensus is now estimated to be 6,216–10,937 in Nairobi and 3,718–8,500 in the Coastal Kenya [19–21]. The national size of the PWID population in Kenya, however, has only been estimated to be 30,000 [22] to 35,000 [4] PWID. Although the population of PWID in Kenya is relatively small, HIV transmission through injection yields a markedly higher HIV incidence rate compared to transmission through heterosexual sex [6]; PWID have two times as high a probability of HIV transmission per risky exposure compared to exposure from casual heterosexual sex [23]. Thus while only an estimated 5.8 % of new HIV infections in Nairobi and 6.1 % on the coast are attributed to PWID, there is great potential for the HIV epidemic to rapidly gain traction in this population [6]. At the time of this study, there were no systematic harm reduction programs for PWID in Kenya. Based on our formative assessment for this study, in Nairobi, PWID typically obtained needles and syringe from pharmacists for about KSH 15–20 (about USD 0.16–0.22). The formative assessment also revealed that PWID face a great deal of stigma from the community as their behavior is criminalized and the community associates drug users with criminal activity since many engage in stealing as a means to support their drug habit. The police often use injection track marks on the body as a basis for arrests. PWID may also be arrested for being in possession of any drug injection paraphernalia.

While limited data exist on characteristics of PWID in Kenya, studies in other countries show PWID commonly engage in high-risk injection behaviors, such as sharing previously used equipment (e.g., needles) [24–27] and also engage in high-risk sexual practices, including exchanging sex for money or drugs [28–34]. Migratory and high-risk sexual behaviors of PWID can act as a bridge to the general population as well [34]. HIV prevalence estimates in Kenya are also limited, but point to highly concentrated pockets of HIV infection within these communities. Previous reports of HIV prevalence in Kenyan PWID range from 31 to 50 % in Nairobi and coastal areas [35–37]; however, these studies relied on convenience sampling or service records, making inferences to the overall PWID population difficult.

This is the first study implemented to provide population-based estimates of characteristics of PWID and the prevalence of HIV and STI in this high-risk population in Nairobi. This information is crucial for guiding HIV prevention programs in Nairobi.

## Methods

### Study Population and Sampling

Between January and March 2011, PWID were recruited for a cross-sectional study using respondent-driven sampling (RDS), an adaptive peer-recruitment sampling method [38–41]. A sample recruited using RDS which meets the underlying assumptions can be used to estimate population parameters. Recruitment was initiated with six ‘seeds’ (initial participants) who were purposively selected in consultation with key informants in the PWID community and diversified on characteristics including sex, age, education, and place where they buy drugs or inject. Seeds were given two recruitment coupons to recruit peers. This process was repeated with subsequent recruits until the research team determined that recruitment was stalling and that only a few PWID could be recruited beyond that point. The number of peers that participants could recruit was limited to keep the number of daily PWID arriving at the study site at a manageable level, encourage long recruitment chains to yield a wider cross-section of the population.

Eligible participants were males and females 18 years and older who reported injecting illicit drugs in the previous 3 months, lived in Nairobi or adjacent urban areas, and were willing to provide written informed consent. Study activities were conducted at the National AIDS and STI Control Programme (NASCOP) voluntary counseling and testing (VCT) center located within the Kenyatta National Hospital compound in Nairobi.

### Survey Data Collection

Face-to-face interviews were conducted in private by trained nurse counselors who used handheld computers. Survey questions included demographics, HIV knowledge, sexual risk and prevention behaviors, drug use, HIV testing history, and experience with violence and discrimination. Upon completion of the behavioral interview, HIV counseling and testing was offered to participants who elected to be tested. An RDS coupon manager software was used to track recruitment and compensation. Participants were compensated KES 400 (approximately USD 4.25) for participating. Participants also received KES 200 (approximately USD 2.15) for each peer they recruited into the study, in addition to KES 200 for transport. Biometric software was used to identify duplicate recruits, confirm correct ownership of a recruit’s coupon, and identify recruits during follow-up. The software creates a unique ID number based on the scan of the participant’s fingerprint, which is immediately deleted after the unique ID is created.

### Laboratory Procedures

Per national guidelines [42], HIV testing was conducted using a parallel algorithm with Determine and Unigold rapid tests (Determine; Abbott Laboratories, Abbott Park, Illinois, USA; Unigold; Trinity Biotech plc, Bray, Ireland), and Bioline (Standard Diagnostics Inc., Gyeonggi-Do, South Korea) as a tiebreaker for discordant results. Those who tested positive were referred to government clinics and some NGO clinics (e.g., NOSET) for further management. For syphilis, rapid plasma reagent (RPR) assays was used for screening, and *Treponema palladium* hemagglutination assay (TPHA) test (Human Diagonistic Worldwide; Wiesbaden, Germany) was used for all positive RPR tests for confirmation. The rapid *C.* *trachomatis* PCR (Roche Amplicor CT/NG test assay, Roche Molecular Diagnostics; Pleasanton, CA) was used for the detection of antigens in urine (men) or vaginal (women) swabs for chlamydia (CT) and gonorrhea (NG). For female respondents only, detection of *T.* *vaginalis* was performed using the InPouch™ system (BioMed Diagnostics, San Jose, California). A vaginal culture was evaluated for bacterial vaginosis and candidiasis using Nugent’s scoring criteria, and the KOH test for candidiasis. For quality assurance of HIV testing, additional HIV testing was conducted off-site. The rapid testing algorithm was repeated at the University of Nairobi Institute of Tropical and Infectious Diseases (UNITID) laboratory for all HIV-positive results and for 5 % of HIV-negative results. PCR was used to resolve any discrepancies. Samples for all STIs were sent and processed at UNITID laboratory. For NG and CT, quality control panels were provided by the Institute of Tropical Medicine (ITM). A panel was tested prior to study implementation and every 4 months after; results were immediately reported to the ITM. For quality control of syphilis, all RPR positive specimens and 5 % of negative specimens were retested with TPHA.

### Data Analysis

RDS Analysis Tool (RDSAT) software version 6.0 was used to produce point estimates and 95 % confidence intervals (CI) [43]. RDSAT adjusts for recruitment patterns and the relative sizes of participants’ networks and theoretically produces unbiased estimates of population characteristics of interest. Multivariable analysis was conducted to determine factors associated with the primary outcome of HIV serostatus. The individualized weights on HIV serostatus were generated in RDSAT, exported to STATA software (Version 11.0, STATA Corp., College Station, TX, USA), and used in the logistic regression analyses with the *pweight* option. We assessed collinearity between injection variables related to the last 1 month and lifetime and found no significant collinearity. Variables associated with HIV infection at *p* < 0.10 level in the bivariate analysis were included in the initial multivariable model. Variables not significant at the 0.05 level were systematically removed from the model using the backwards stepwise method. The final model includes variables that were associated with HIV infection at *p* < 0.05 level or were considered to be salient confounders, such as age.

### Variable Definitions

Poly-drug use was defined as concurrent drug use (injected or non-injected) with at least one other drug injected in the past 1 month. Sharing drug injection equipment includes sharing of dropper, water, bleach, and other cleaning agents. The variable “risky injection practices” is a composite variable that includes using needles/syringe after someone else used it (referred to hereon as “receptive syringe sharing”), using a pre-filled needle/syringe, front- or back-loading injections (a method of sharing drugs by transferring contents from one syringe to another), sharing of preparation water, sharing of other injections equipment, or drawing drugs from a common container. A regular partner was defined as someone with whom the respondent had an ongoing or long-term intimate sexual relationship; it included live-in partners and spouses. A casual partner was defined as a partner with whom the respondent did not have an ongoing or intimate sexual relationship, and includes one-time encounters. Commercial partners were those who the respondent paid for sex or paid the respondent for sex with money, goods or services.

### Ethical Approval

The study protocol was approved by the Kenyatta National Hospital Ethics and Research Committee, the Population Council Institutional Review Board, and the Global AIDS Program at the Centers for Disease Control and Prevention (CDC).

## Results

**Fig. 1:**
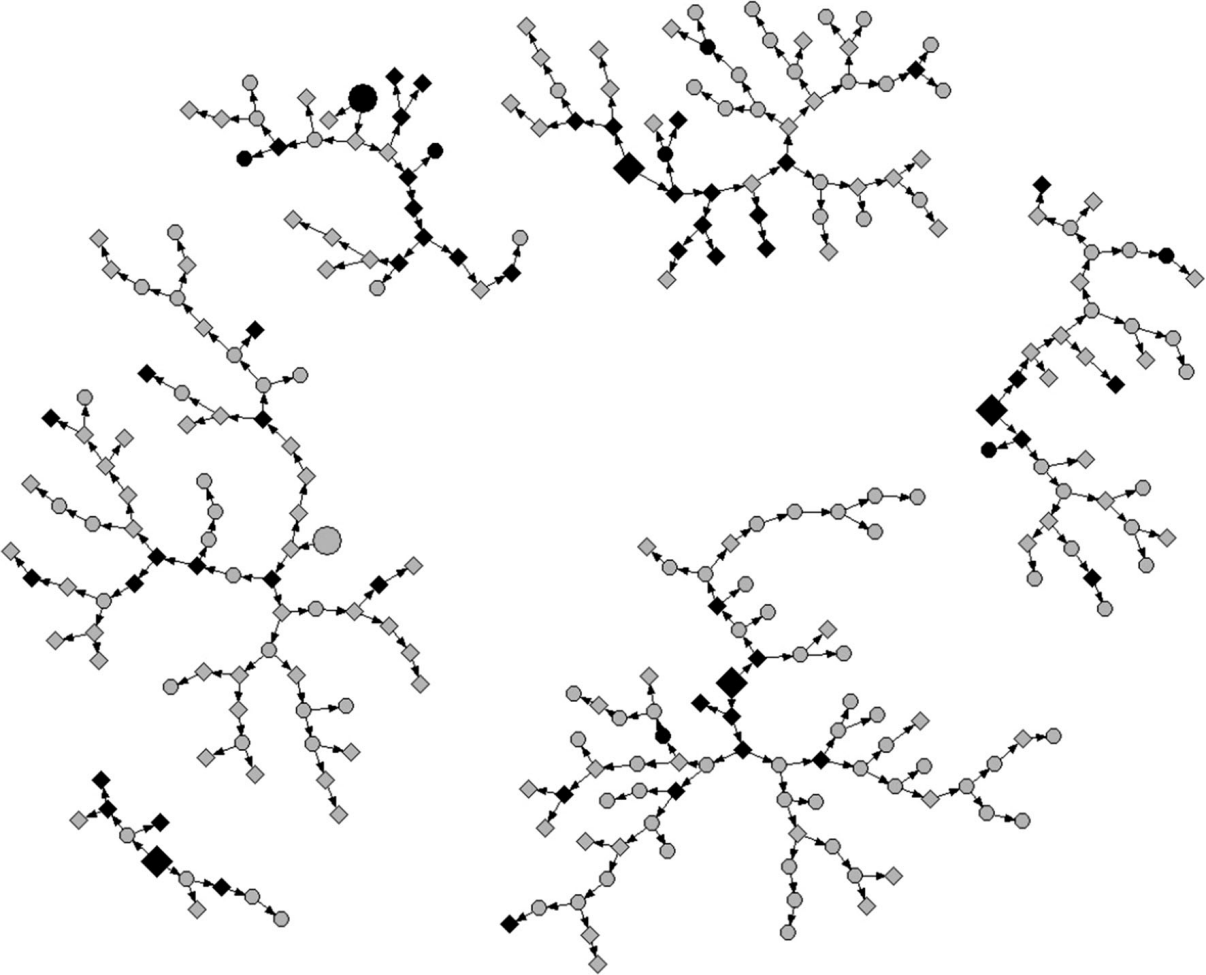
Network diagram of PWID recruitment chain referral in Nairobi, 2011 (*N* = 275), by HIV serostatus and lifetime sharing of needle or syringe. *Larger shapes* depict seed participants (*n* = 6); *smaller shapes* are recruited respondents (*n* = 269). *Gray* HIV-seronegative respondents (*n* = 212; 1 seeds). *Black* HIV-seropositive respondents (*n* = 57; 5 seeds). *Square* Ever receptive syringe sharing (*n* = 151; 4 seeds). *Circle* Never receptive syringe sharing (*n* = 118; 2 seeds)

Of the 352 PWID screened for eligibility between January and March 2011, six were seed participants and 77 were found to be ineligible, yielding an analytic sample of 269 non-seed participants who completed the survey and testing. Figure 1 illustrates the network recruitment chains. Demographic characteristics and risk behaviors are presented in Table 1. The median age of PWID was 31 years. In adjusted analysis, the majority were male (92.5 %), 16.9 % were currently married and 58.6 % were previously married. PWID mainly earned money through informal or irregular employment, with only 11.4 % of the population having formal employment. Nearly one out of five (18.8 %) earned income through illegal activities and/or sex work.

**Table 1:** Crude and RDS-weighted demographic characteristics of PWID in Nairobi, 2011 (*N* = 269)

Variable	Crude % (*n*)	Weighted % (95 % CI)
Age (median 31, IQR 27–37)		
18–24	10.0 (27)	9.9 (5.2–15.6)
25–29	26.0 (70)	23.7 (17.0–30.0)
30–34	27.9 (75)	20.2 (22.1–38.9)
35 years or older	36.1 (97)	36.2 (28.9–44.5)
Gender		
Male	92.2 (248)	92.5 (86.4–97.5)
Female	7.8 (21)	7.5 (2.5–13.6)
Education		
None/incomplete primary	47.6 (128)	51.2 (42.5–59.9)
Completed primary	17.8 (48)	17.2 (11.6–23.3)
Some secondary or higher	34.6 (93)	31.5 (24.0–39.4)
Current marital status		
Single, never been married	27.5 (74)	24.4 (17.1–32.0)
Single, formerly married	54.3 (146)	58.6 (50.6–66.5)
Currently married	18.2 (49)	16.9 (11.3–23.9)
Kenyan		
Kenyan	98.5 (265)	97.9 (94.9–100)
Non-Kenyan	1.5 (4)	2.1 (0–5.1)
Employment		
No income/not employed	4.46 (12)	3.2 (1.4–5.9)
Skilled labor/sales/professional	11.5 (31)	11.4 (6.0–17.0)
Casual laborer/scavenger	34.9 (94)	31.4 (23.7–37.7)
Transport worker	23.1 (62)	22.3 (16.0–31.4)
Illegal activity/sex work	17.1 (46)	18.8 (12.1–26.6)
Other	8.9 (24)	12.8 (7.2–19.3)

### Drug Injection Behaviors

Table 2 presents drug using and sexual risk indicators. While PWID initiated drug use at a median age of 18 years, the median age at first drug *injection* use was 33 years. The most commonly drug first used in this population of PWID was marijuana (57.2 %) and first drug injected was white heroin (84.3 %), which is the drug most commonly injected at the time of the study (by 96.5 % of participants). Over 40 % of PWID in Nairobi were recent initiators of injection drug use; an estimated 21.3 % started injecting 5 or more years ago.

**Table 2:** Crude and RDS-weighted behavioral and HIV prevalence variables of PWID in Nairobi, 2011 (*N* = 269)

Variable	Crude % (*n*)	Weighted % (95 % CI)
Median age at first drug use [IQR]	18 [13–20]	
Time since first illicit drug use		
<10 years	30.7 (82)	30.6 (22.9–38.7)
10–<20 years	49.4 (132)	53.2 (44.4–61.8)
20 years or more	19.9 (53)	16.2 (11.1–22.1)
First illicit drug used		
Marijuana	62.1 (167)	57.2 (48.5–66.9)
Heroin	20.8 (56)	22.5 (14.9–29.9)
Other	17.1 (46)	20.3 (14.9–29.9)
Median age at first injection [IQR]	33 [30–35]	
First illicit drug injected		
White heroin	84.4 (227)	84.3 (79.2–89.9)
Brown heroin	14.9 (40)	15.5 (9.9–20.6)
Other	0.7 (2)	0.2 (0.1–0.5)
Time since first injection		
≤6 months	35.4 (95)	43.3 (33.6–53.9)
7 months to <5 years	40.3 (108)	35.4 (26.9–44.5)
5 years or more	24.3 (65)	21.3 (14.2–28.4)
Type of drug injected currently		
White heroin	97.0 (261)	96.5 (93.2–99.0)
Other	3.0 (8)	3.5 (1.0–6.8)
Poly-drug, use in past 1 month^a^		
Marijuana	66.5 (179)	64.5 (56.1–72.4)
Khat	10.8 (29)	14.8 (8.9–21.3)
Cocaine	3.7 (10)	5.7 (1.4–11.3)
Tranquilizers	58.0 (156)	50.1 (41.8–58.6)
Drug injection behaviors		
Injection frequency in the past 1 month		
Everyday	79.8 (214)	77.3 (70.6–84.8)
Less than everyday	20.2 (54)	22.7 (15.2–29.4)
Most common location where PWID inject		
At base where drugs were bought^b^	67.7 (182)	60.6 (52.3–67.5)
Home	34.6 (93)	24.9 (19.2–31.3)
Street or park	32.3 (87)	32.6 (25.5–39.9)
In dealer/peddler’s home	9.7 (26)	13.7 (8.6–19.3)
Any abandoned building	8.2 (22)	7.5 (3.8–11.3)
Injecting risk behaviors in the past 1 month		
Receptive syringe sharing^c^	47.0 (126)	47.4 (38.8–55.9)
Used pre-filled needle/syringe	29.1 (78)	33.2 (25.4–41.3)
Front- or back-loaded needle/syringe	46.0 (123)	46.3 (38.1–55.0)
Shared water used to prepare drugs	59.0 (158)	57.1 (48.4–65.2)
Shared equipment^c^	58.2 (156)	56.9 (48.7–64.6)
Drew drugs from common container	38.1 (102)	37.9 (29.7–46.7)
Lent needle/syringe	52.6 (141)	50.0 (41.6–57.9)
Risky Injection in the past 1 month^d^	71.6 (192)	67.3 (58.9–75.6)
Lifetime injecting risk behaviors		
Ever receptive syringe sharing^c^	56.1 (151)	53.8 (45.4–62.1)
Ever used pre-filled needle/syringe	39.9 (107)	41.8 (33.9–50.0)
Ever shared water used to prepare drugs	61.7 (166)	63.4 (55.4–70.6)
Ever shared equipment^d^	65.1 (175)	64.4 (56.3–71.9)
Ever drew drugs from common container	51.3 (138)	48.6 (40.6–57.3)
Risky injection ever in lifetime^e^	78.8 (212)	80.0 (73.9–85.5)
Sexual behaviors		
Sexually active in the past 1 month	39.0 (105)	40.7 (32.3–49.5)
Partner type in the past 1 month		
No partner	60.6 (163)	59.0 (50.4–67.2)
Regular^f^	28.3 (76)	29.5 (21.7–38.0)
Casual^f^	4.8 (13)	5.4 (1.4–10.8)
Commercial^f^	6.3 (17)	6.1 (2.8–10.1)
Condom use in the past 1 month among sexually active PWID (*n* = 105)		
Always	17.1 (18)	20.8 (8.5–36.1)
Sometimes/never	82.9 (87)	79.2 (63.9–91.5)
Multiple sex partners in past 12 months		
None or 1 partner	76.2 (205)	77.0 (68.6–83.8)
More than one partner	23.8 (64)	23.0 (16.2–31.4)
Last female sex partner ever injected drugs (among sexually active male PWID) (*n* = 86)		
Yes	36.1 (31)	36.3 (26.6–69.2)
No	63.9 (55)	63.7 (30.8–73.4)
Knows HIV status (from prior testing)		
Yes	80.0 (212)	78.9 (72.7–85.8)
No	20.0 (53)	21.1 (14.2–27.3)
HIV prevalence (All)	21.2 (57)	18.7 (12.3–26.7)
Gender		
Male	19.4 (48)	15.4 (10.5–23.2)
Female	42.9 (9)	60.7 (14.7–87.2)
Age		
≤34 years	26.7 (46)	22.1 (13.3–33.2)
35 years or older	11.3 (11)	13.0 (4.8–23.3)
STI prevalence		
Syphilis	1.5 (4)	1.7 (0.2–6.0)
Gonorrhea	1.1 (3)	1.5 (0.1–4.9)
Chlamydia	3.4 (9)	4.2 (1.2–7.8)

^a^Concurrent drug use (injected or non-injected) with at least one other drug injected in the past 1 month

^b^A base is typically a public open outdoor space where PWID buy drugs. PWID may inject there as well

^c^Receptive syringe sharing refers to using a needle/syringe used by someone else

^d^Sharing equipment includes sharing of dropper, water, bleach, and other cleaning agents

^e^Risky injection practices variable is a composite variable that includes using needles/syringe after someone else used, using a pre-filled needle/syringe, front- or back-loading injections, sharing of preparation water, sharing of other injections equipment, or drawing drugs from a common container

^f^Regular partner: Someone with whom the respondent had an ongoing or long-term intimate sexual relationship; it included live-in partners and spouses. Casual partner: A partner with whom the respondent did not have an ongoing or intimate sexual relationship, and includes one-time encounters. Commercial partner: A partner who the respondent paid for sex or paid the respondent for sex with money, goods or services

Recent poly-drug use was common with almost two-thirds using marijuana and 50.1 % using tranquilizers in addition to heroin in the most recent month. The majority (77.3 %) injected daily. PWID most commonly injected where they buy drugs (60.6 %) or in the street or parks (32.6 %); 24.9 % injected at home.

PWID engaged in various high-risk injection practices in the most recent month including receptive syringe sharing (47.4 %), pre-filled needles and syringes (33.2 %), front- or back-loading (46.3 %), sharing preparation water (57.1 %), sharing equipment (56.9 %), and drawing drugs from a common container (37.9 %). Overall, an estimated 67.3 % engaged in at least one of these risky injection practices in a typical month, and 80.0 % are estimated to have ever engaged in any of these practices in their lifetime. An estimated one-half lent their needles or syringe to someone else in the most recent month. Of these (*n* = 141), 23.7 % were HIV-positive and 16.7 % knew that they were HIV-positive. (Data not shown)

### Sexual Risk Behaviors

While over one-half (59.0 %) did not have any sex partners in the past month, 29.5 % had a regular sex partner (Table 2). Having casual (5.4 %) and commercial (6.1 %) partners in the past month was less common. Among female participants only (*n* = 21), however, selling of sex was reported by 5 of 21 respondents (23.8 %, unadjusted, data not shown). Consistent condom use with any partner was not common; only 20.8 % of sexually active PWID used condom consistently in the most recent month. Nearly one-quarter (23.0 %) had more than one partner in the prior 12 months. Over one-half (63.7 %) of males who inject drugs indicated that their last female sex partner has never injected drugs.

### HIV Testing and HIV and STI Prevalence

A majority had previously tested for HIV (78.9 %). HIV prevalence was 18.7 % (95 % CI 12.3–26.7). The prevalence of STI was lower than HIV prevalence [syphilis: 1.7 % (95 % CI 0.2–6.0); gonorrhea: 1.5 % (95 % CI 0.1–4.9); and Chlamydia: 4.2 % (95 % CI 1.2–7.8)]. Although the confidence interval was wide and overlapped that of the males, females who inject drugs had a much higher estimated HIV prevalence (60.7 %; 95 % CI 14.7–87.2) compared to males who inject drugs (15.4 %; 95 % CI 10.5–23.2). Approximately one-quarter (28.8 %) of those who tested HIV-positive were unaware that they were infected. Female PWID were tested for Trichomoniasis and Bacterial vaginosis; of the 21 women, 8 (38.1 %) were found positive for each of these infections. (Data not shown)

### Factors Associated with HIV Infection

Table 3 shows the bivariate and multivariable analyses of the association between HIV-positive status and demographic and behavioral characteristics among PWID. Bivariate analysis indicated that HIV-positive status was significantly associated with being female (OR, 8.7; *p* = 0.003) and longer time since first injection (OR, 4.6; *p* = 0.001). Additionally, risky injecting practices in the past 1 month (using a pre-filled needle or syringe, sharing water used to prepare drugs, and drawing drugs from a common container) as well as lifetime risky injection practices (receptive syringe sharing, using a pre-filled needle or syringe, sharing water used to prepare drugs, and sharing equipment) were significantly associated with HIV-positive status at the *p* < 0.05 level.

**Table 3:** RDS-weighted bivariate and multivariate associations between demographic and selected variables and HIV infection among PWID, Nairobi, 2011 (*N* = 269)

Variable	OR	*p* value	aOR^a^	95 % CI	*p* value
Demographic characteristics					
Age					
≤34 years	1		1		
35 years or older	0.5	0.117	0.4	0.1–1.1	0.072
Gender					
Male	1		1		
Female	8.7	0.003	3.5	1.0–12.2	0.048
Education					
Primary or less	1				
Secondary or higher	1.1	0.790			
Injection behaviors (lifetime)					
Time since first injection					
0–4 years	1		1		
5 years or more	4.6	0.001	4.3	1.7–10.9	0.002
Risky injection ever in lifetime^b^	3.8	0.050			
Receptive syringe sharing^c^	7.9	<0.0001	6.2	2.2–17.6	0.001
Used pre-filled needle/syringe	5.1	<0.0001			
Front- or back-loaded needle/syringe					
Shared water used to prepare drugs	3.6	0.014			
Shared equipment^d^	3.9	0.006			
Drew drugs from common container	1.8	0.203			
Injection behaviors (past 1 month)					
Risky injection in the past 1 month^b^	2.0	0.127			
Receptive syringe sharing^c^	2.2	0.063			
Used pre-filled needle/syringe	2.5	0.033			
Front- or back-loaded needle/syringe	2.3	0.052			
Shared water used to prepare drugs	2.6	0.025			
Shared equipment^d^	2.1	0.101			
Drew drugs from common container	3.1	0.008			

^a^ Adjusted for all other variables in the model

^b^ Risky injection practices include using needles/syringe after someone else used, using a pre-filled needle/syringe, front- or back-loading injections, sharing of preparation water, sharing of other injections equipment such as spoons or cookers, or drawing drugs from a common container

^c^ Receptive syringe sharing refers to using a needle/syringe used by someone else

^d^ Sharing equipment includes sharing of dropper, water, bleach, and other cleaning agents

In the final multivariable model (Table 3), HIV infection was associated with being female (aOR, 3.5; *p* = 0.048), having first injected drugs 5 or more years ago (aOR, 4.3; *p* = 0.002), and ever having practiced receptive syringe sharing (aOR, 6.2; *p* = 0.001). While Fig. 1 reflects only peer recruitment, and not necessarily actual needle-sharing relationships, it does show some clustering of PWID by HIV serostatus and riskier participants (i.e., those who have ever engaged in receptive syringe sharing). Specifically, 23 HIV-positive respondents who have ever engaged in receptive syringe sharing were recruited into the study by a participant with the same characteristics (HIV-positive, ever engaged in receptive sharing) and only 5 HIV-positive respondents were recruited into the study by a participant with a different receptive syringe sharing profile.

## Discussion

This study is the first to report population-based prevalence of HIV, STIs, and risk behaviors in PWID in Nairobi, Kenya. Our findings indicate high seroprevalence of HIV and high levels of risky injection behaviors. Moreover, we found that HIV infection was independently associated with lifetime practice of using previously used needles and syringes and longer time since first injection.

Our findings of 18.7 % (95 % CI 12.3–26.7) HIV prevalence is similar to that found in Zanzibar (16.1 %; 95 % CI 11.3–21.1), which also used RDS [44]. However, our HIV prevalence is slightly lower than that reported in a previous study of PWID in Nairobi, which found HIV prevalence of 36.3 % [45], as well as those reported in other east African countries: 47 % in Mauritius, 27–58 % in Tanzania, and 26 % in Zanzibar [15, 46–48]. However, these studies were not based on a probability-based sample with the exception of Mauritius. Another recent study of PWID in Nairobi was implemented using RDS methods shortly after this study, and reported an equivalent HIV prevalence of 18.3 % [21]. Thus the similarity in HIV prevalence estimates from these two studies illustrates the reliability of the RDS method when conducted from different central locations in the Nairobi area.

The HIV prevalence among male PWID (15.4 %) in Nairobi is 3.5 times as high as that of men aged 15–49 years (4.4 %) in the general population [17]. Immediate comprehensive prevention programming, including harm-reduction interventions, is warranted in order to prevent transmission of HIV among PWID and to their non-injecting sex partners. In fact, our data indicated that over one-half of males who inject drugs in Nairobi who were sexually active had non-injecting female partners. Thus, this bridge from PWID to the non-injecting population has a very high potential for fueling the pandemic in the general, non-injecting population. Therefore, by not fully addressing HIV infection in key populations with high HIV prevalence, such as in PWID, current efforts to reduce HIV spread in the general population could be compromised.

The small number of female PWID in this survey is similar to other studies conducted in African settings [16, 49–51]. Although the estimate may be imprecise due to the small sample size, there is indication that the HIV prevalence among women may be higher than that of the men (60.7 vs. 15.4 %). Other African studies have documented HIV prevalence to be 2–10 times as high among female as among male PWID [16, 47, 52–54]. Furthermore, in our final multivariable model, being female was borderline significantly associated with HIV infection. Additional research is needed to estimate the population size of female PWID in Nairobi and to better understand risk behaviors and access to prevention services in this sup-population to inform prevention programs targeting PWID that may include women.

Based on our estimated HIV prevalence of 18.7 % and the estimated PWID population size range of 6,216–10,937 in Nairobi [19], there are an estimated 1,162–2,045 HIV-positive PWID in Nairobi. One-quarter of those testing HIV-positive did not know they were infected; thus, there is need to increase HIV testing rates among PWID. HIV-positive PWID will also need to be linked to HIV care and treatment services. HIV treatment can serve as prevention when combined with other HIV prevention efforts [55]. Therefore, programs should ensure that PWID are not denied HIV treatment due to stigma and discrimination against PWID.

Furthermore, in the multivariable model of factors associated with HIV infection, longer time since first injection (5 years or longer) was a strong independent factor, which represents increased risk of infection related to the cumulative exposure to injection-related risk over time. This is similar to other studies which found higher HIV prevalence among longer term PWID [56, 57]. We also found that a high proportion (43 %) of PWID in Nairobi initiated drug injection within the past 6 months, nearly 80 % initiated in the past 5 years, and many are daily injectors. Prospective studies, conducted at the height of the HIV epidemics in other countries, found higher HIV incidence among recent-onset PWID [30, 57–59]. As such, evidence-based prevention programs tailored toward PWID would benefit from targeting this population soon after injection initiation to establish safer injection practices early and to stop drug use while the habit is still new. Substance abuse counseling and treatment, including medication assisted treatment, should be available to all PWID to support reduction and eventual cessation in drug injection. Programs targeted at recently-initiated PWID should also consider that many of them are daily injectors. It is not uncommon for recently-initiated PWID to inject daily. Nearly all PWID in our study injected heroin, which is highly addictive and typically requires daily use to prevent withdrawal. Based on our formative assessment, heroin in Nairobi is high in purity which makes it possible to snort. It is possible that PWID in our study began using heroin through snorting and thus may have already been addicted to heroin and likely using daily when they transitioned to injecting.

Similar to other studies conducted in sub-Saharan Africa [24–27], PWID in Nairobi reported high levels of both lifetime and past-month risky injection behaviors, including receptive syringe sharing, front- or back-loading needle/syringes, and sharing water used to prepare drugs. Furthermore, ever receptive syringe sharing remained independently associated with HIV infection. Equally alarming is the high proportion of PWID in Nairobi lending their needles/syringes to others; of those who did so, nearly one-quarter were HIV-positive. Together these findings highlight the urgent need to strengthen current outreach efforts to increase personal awareness of risk and to decrease sharing of injection equipment. Such efforts should be coupled with increased access to sterile injection equipment. Needle and syringe programs, as part of a comprehensive prevention approach for PWID, have been found to be an effective and cost-effective approach to reducing injection-related risk behaviors and the spread of HIV, without negative consequences [60, 61], and may be an important venue to reach HIV-positive PWID for linkage to HIV care and treatment.

Poly-drug use, particularly heroin in conjunction with tranquilizers, was found to be common among PWID in Nairobi. Poly-drug use can be fatal, especially if heroin is used in combination with benzodiazepines (tranquilizers) [62, 63]. Such combination also has implications for drug abuse treatment as methadone treatment may not be as effective for heroin users if the tranquilizer use is also not addressed [62–64]. If medically assisted therapy is to be initiated in Kenya, guidelines will be needed to address issues related to treating poly-drug use. Additionally, studies have found that opiate users who also used benzodiazepines were more likely to have psychological vulnerabilities (i.e., depression, self-harm attempts) [63, 65, 66]. Prevention and treatment programs need to address these psychological co-morbidities.

Nairobi PWID indicated that they injected drugs where they bought the drugs or on the street, which suggests that outreach should be used to target prevention activities in these areas. PWID typically congregate at these ‘bases’, which are typically outdoor public areas where drug dealers sell drugs and are well-known by PWID. Even PWID who want to remain anonymous come to the bases to buy drugs; however, they typically stay in their cars and do not linger to inject at the base. Thus, outreach to these ‘hotspots’ can be ideal for reaching a variety of PWID with prevention activities. Outreach is one of the most commonly used and more effective strategies for reaching PWID with harm reduction interventions [67–70].

Sexual activity was fairly low among PWID; only four out of ten PWID reported being sexually active in the past 1 month. Few PWID engaged in casual and commercial sexual activity. Most of the sexually active PWID had regular partners. However, the majority did not use condoms consistently in these sexual partnerships, thus putting PWID and their regular partners at risk for HIV and other STIs. These findings are suggestive that risky injecting behaviors rather than sexual practices may be the most important factor contributing to HIV transmission among this population. Although drug-injecting behaviors should remain the focus of any harm-reduction programs, condom use, particularly within regular partnerships, should be part of a comprehensive HIV prevention program for PWID.

Some limitations should be mentioned. The actual sample size was lower than our target sample size due to the time and resources available. Low recruitment may have been due in part to the distance of the study site from drug-using locations. Recruitment remained low despite attempts to offer transport to the study site from drug-using locations. Although the relatively small sample size resulted in wider CI for some estimates, particularly HIV prevalence in female PWID, the estimates still provide useful information for programs and policies as this was a rigorously conducted population-based study. While it is possible that fewer females participated in the study due to the study’s STI testing procedures such as a vaginal swab, it is likely to be minimal since they were free to decline this procedure. The study’s small proportion of females is also in line with other studies of PWID in the African region. Further research is needed particularly for female PWID. Future studies with PWID should also make an effort to recruit middle- and upper-class PWID. Additionally, the high proportion of recently initiated PWID may be a result of sampling bias that RDS is not able to account for or social desirability bias (if participants perceive longer term use as negative). However, high proportions of recently initiated PWID have been observed in other surveillance surveys with PWID. In Nigeria, the median time since first injection was 2–7 years across six states [16]. Lastly, assumptions for RDS must be met in order to arrive at an unbiased estimate. Based on our formative assessment for this survey, two of the four assumptions were likely met (i.e., the target population know each other as members of the population in question and networks form one single large component). The assumption that participants report network size accurately was likely met given that the mean (26), median (10), and the interquartile range (5 and 20) are reasonable. The last assumption (i.e., random recruitment of peers) is difficult to assess within the context of a surveillance survey.

## Conclusion

Our findings indicate that given the high HIV prevalence and the high levels of risky injection practices among PWID in Nairobi, there is a potential to see increases in HIV prevalence in this population. In addition, more than one-half of sexually active PWID had non-injecting sex partners, thus HIV infection among PWID may continue to fuel the HIV epidemic in the general, non-injecting population. Thus, comprehensive, harm-reduction programs tailored toward PWID and their sex partners need to be implemented in full force. Comprehensive harm reduction programs for PWID include access to sterile injection equipment, drug dependence treatment, ART for those who are HIV positive, HIV counseling and testing, prevention and treatment of STIs, prevention, vaccination, and treatment of viral hepatitis, prevention and treatment of TB, and risk reduction information for PWID and their sex partners, and condom distribution for PWID and their sex partners [71]. There is evidence of harm-reduction programs reducing risky injection behaviors, and countries that have implemented harm-reduction programs have seen declines in new drug-related HIV infections [72, 73]. Modeling exercises show that if key interventions such as HIV counseling and testing, needle exchange and drug treatment such as medication-assisted treatment are offered along with needed expansion of ART, approximately 2,000 new HIV infections could be averted among PWID between 2012 and 2015 throughout Kenya, which represents a 56 % reduction compared to the status quo scenario [4]. There is evidence that PWID can adhere to ART regimen with supportive interventions such as psychosocial support, medication assisted therapy for drug dependence, directly administered ART, and nurse-delivered interventions [74–76].

The Kenyan NASCOP, Kenya AIDs and NGOs Consortium through the support of AIDS Alliance (KANCO), and the Kenya Red Cross through Global Fund are initiating harm-reduction interventions per the WHO and Kenya’s guidelines including needle and syringe exchange programs, medication assisted therapy with both powdered and liquid methadone, Hepatitis C screening, Hepatitis B screening and vaccination, and opiate overdose management with Naloxone in Nairobi, Mombasa, and Kilifi counties [4, 77]. The program has also included the provision of two different sizes of needles (23G/1 ml and 28G/3 ml) as some prefer smaller gauge needles in order to reduce the scars on their body [77]. The harm reduction program is also currently working with the law enforcement sector to sensitize them on the issues related to PWID and harm reduction. Research and evaluations are needed to determine the effectiveness of these programs in reducing the impact of HIV in this key population as well as the general population and document lessons learned from the implementation. Integrated bio-behavioral surveillance (BBS) should continue in order to monitor sexual and injecting behaviors among PWID, their access to prevention and harm reduction programs, and effect of these programs on trends in HIV prevalence in this population. The BBS should also be expanded to other areas in Kenya with drug injection problem, particularly in the coastal areas.
